# Changes of Mind after Movement Onset Depend on the State of the Motor System

**DOI:** 10.1523/ENEURO.0174-21.2021

**Published:** 2021-12-10

**Authors:** Ignasi Cos, Giovanni Pezzulo, Paul Cisek

**Affiliations:** 1 Facultat de Matemàtica i Informàtica, Universitat de Barcelona, 08007 Barcelona, Spain; 2 Serra-Húnter Fellow Programme 08010 Barcelona, Spain; 3 Institute of Cognitive Sciences and Technologies–National Research Council, 00185 Rome, Italy; 4 Department of Neuroscience, University of Montréal, Montréal, Québec H3T 1J, Canada

**Keywords:** change of mind, decision-making, KINARM, motor system, reward

## Abstract

Decision-making is traditionally described as a cognitive process of deliberation followed by commitment to an action choice, preceding the planning and execution of the chosen action. However, this is challenged by recent data suggesting that during situated decisions, multiple options are specified simultaneously and compete in premotor cortical areas for selection and execution. Previous studies focused on the competition during planning and left unaddressed the dynamics of decisions during movement. Does deliberation extend into the execution phase? Are nonselected options still considered? Here we studied a decision-making task in which human participants were instructed to select a reaching path trajectory from an origin to a rectangular target, where reward was distributed nonuniformly at the target. Critically, we applied mechanical perturbations to the arm during movement to study under which conditions such perturbations produce changes of mind. Our results show that participants initially selected the direction of movement toward the highest reward region and changed their mind most frequently when the two choices offered the same reward, showing that deliberation continues and follows cost–benefit considerations during movement. Furthermore, changes of mind were dependent on the intensity of the perturbation and the current state of the motor system, including velocity and distance to targets. Although reward remains most relevant, our results indicate that the state of the motor system when the perturbation occurs is a crucial determinant of changes of mind.

## Significance Statement

Our study provides supporting evidence for the notion that deliberation during decision-making continues after movement onset because unselected potential actions are not completely suppressed or discarded. From a neurophysiological perspective, our findings suggest that the competition between actions continues during movement, possibly because the initially unselected neuronal population retains subthreshold activation, which enables them to take control afterward. Furthermore, our findings also suggest that decision-makers have a variable degree of commitment to their initial choice, which depends on the relative reward of the offers and on the state of the motor system. The commitment is stronger if initially leading to a high reward, and changes of mind are more frequent at slow speed and when close to the alternate target.

## Introduction

Decision-making has been traditionally described as a cognitive process, which is completed before the preparation and execution of the action that reports the choice (Newell and Simon, 1972). However, this serial model was developed for laboratory decisions, where options are fixed and actions can be executed almost instantaneously. By contrast, the decision systems of the brain plausibly evolved to deal with situated decisions—for example, a lioness deciding which gazelle to chase—that pose different adaptive challenges (Filimon et al., 2013; Cisek and Pastor-Bernier, 2014; Ratcliffe and Newport, 2017; Wispinski et al., 2020; Ozbagci et al., 2021).

Waiting to reach a decision is rarely possible in situated decisions. Most often, a movement must be started before completely making up your mind, not to miss important opportunities. This may be better fulfilled by brain architectures where decision and motor systems are more intertwined than traditionally assumed, with motor regions engaged early on during deliberation and possibly participating of the decision itself. Experimental evidence revealed that premotor cortex and superior colliculus may encode several competing potential actions before movement, and that the decision process can be characterized as a competition (McPeek and Keller, 2002; Cisek and Kalaska, 2010). This suggests that decisions may be performed by neural areas including sensorimotor regions, rather than being confined to prefrontal areas (Ghez et al., 1997; Cisek, 2012; Kubanek and Snyder, 2015; Carroll et al., 2019).

Involving motor cortices in decisions addresses another challenge of situated decisions: their continuous nature, since actions take time to complete. Furthermore, the environment is nonstationary and key determinants such as the geometric arrangement of targets (e.g., the distance to the prey) and action costs (e.g., energy expenditure to reach a moving target; Morel et al., 2017) can change continuously as the action unfolds. Furthermore, new opportunities may become available or unavailable (e.g., a novel prey can appear; Diamond et al., 2017; Michalski et al., 2020). Accordingly, animals have to continuously re-evaluate their choices after movement onset, rather than committing rigidly to their initial decisions (Lepora and Pezzulo, 2015). Recent studies have shown that movements may be initiated before the decision is complete (Wolpert and Landy, 2012), to later be revised, sometimes causing “changes of mind” (Resulaj et al., 2009; Song and Nakayama, 2009; Barca and Pezzulo, 2012).

In sum, this body of evidence suggests that a situated decision may be neurally implemented as a continuous competition between potential actions, and that spontaneous adjustments and “changes of mind” may occur after movement onset.

Interestingly, it has been shown that changes of mind are not just spontaneous but can also be triggered externally, by sudden target jumps, perturbations to the motor apparatus, or changes of the environment (Nashed et al., 2012, 2014; Burk et al., 2014; Atiya et al., 2020; Marti-Marca et al., 2020). However, it is unclear whether these “externally triggered” changes of mind reflect truly deliberative processes based on cost–benefit considerations (Shadmehr et al., 2010; Rigoux and Guigon, 2012; Taniai and Nishii, 2015; Carroll et al., 2019) or are simpler motor reflexes. Furthermore, if these changes of mind reflect deliberation, what information is considered? If they are influenced by economic variables such as the reward (R) of the nonselected offer, then they should occur less often when the reward of the nonselected offer is lower than the selected option. If they take into account the momentary state of the motor system, then they should occur less often if the perturbation happens when the state of the motor system favors the selected offer (i.e., when one is close to the selected target and/or moving quickly toward it).

To investigate these questions, we designed a reward-driven reach decision task in which movements were sometimes perturbed and predicted that changes of mind should occur more often with strong and early perturbations, when actions are slower, and when the arm position is further away from the initially selected target.

## Materials and Methods

### Participants

A total of 16 subjects (6 males, 10 females; age range, 21–36 years; all right hand dominant) participated in the experimental task. All subjects were neurologically healthy, had normal or corrected-to-normal vision, were naive as to the purpose of the study, and gave informed consent before participating. The study was approved by the local Human Research Ethics Committee and were conducted in accordance with the committee’s ethical standards.

### Task apparatus

During the experimental session, the participants were seated facing the projection system with the right arm supported in a horizontal plane by the KINARM robotic exoskeleton (BKIN Technologies). The KINARM permits elbow and shoulder movements on the horizontal plane, as well as controlled mechanical perturbations to the upper and lower arm sections (Scott, 1999). The display of cues and hand position feedback were presented to the subject by projection onto a mirror, and the arm and hand were occluded and never visible during the experiment. Custom-written software controlled the stimulus presentation and task data collection of shoulder and elbow kinematic and kinetic variables at 1000 Hz. Data from each session was transferred to a MySQL Community Server database (Oracle) for further analysis with custom-designed MATLAB scripts (MathWorks).

### Behavioral task

To determine whether changes of mind take movement-related factors into account, we designed a reward-driven decision-making task in which human participants had to perform a planar movement from an origin cue to a wide rectangular target, in which the reward for each trial depended on where on that target the end point landed. The distribution of rewards was bimodal, with peaks at the extremes and zero in the center.

**Figure 1.**
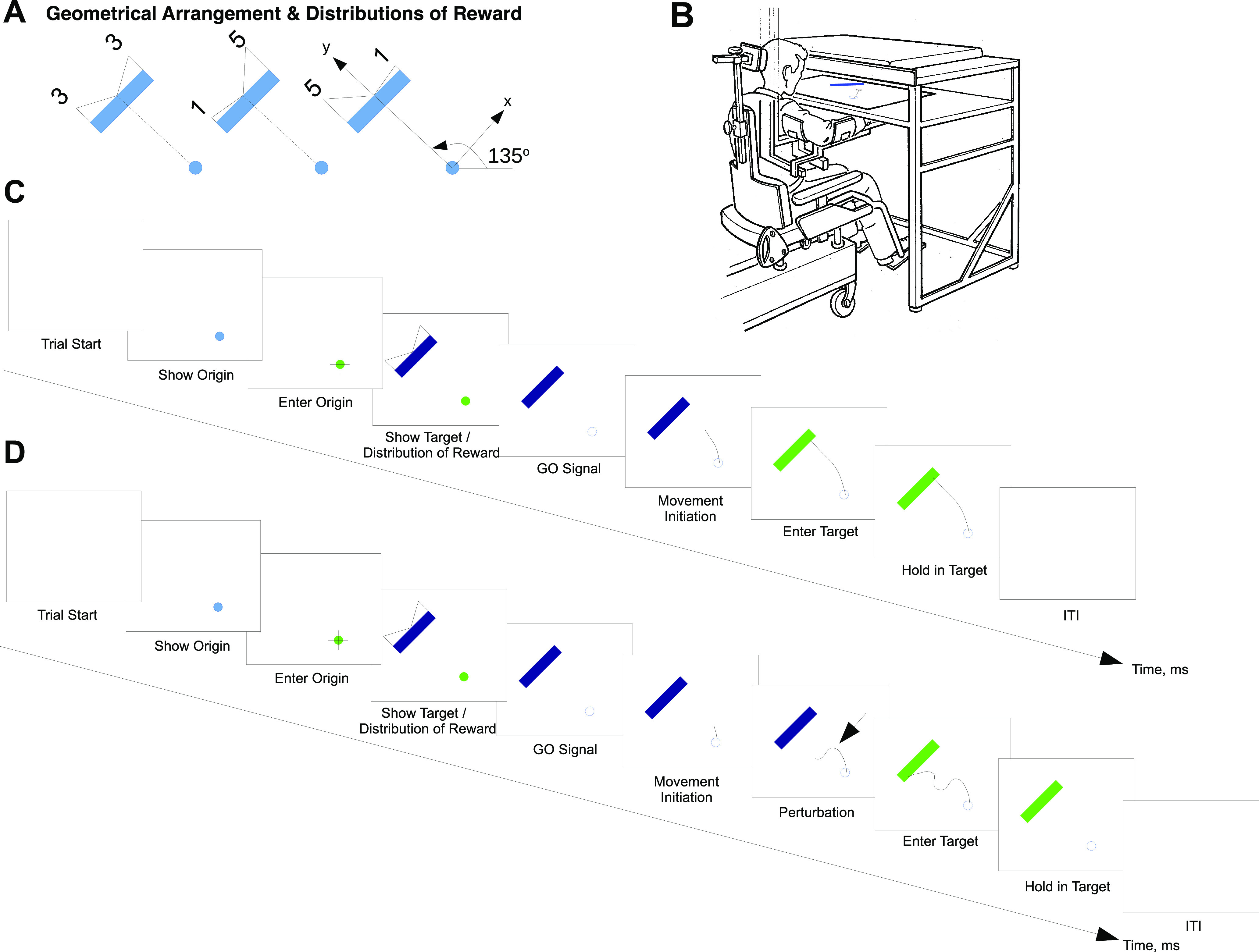
***A***, Geometrical arrangements of the stimuli on different trials consist of a circular origin cue (1 cm) and a rectangular target (width, 10 cm; depth, 1 cm), placed ∼15 cm away from the origin at an orientation of 135°. We show the three distributions of reward, from left to right: 3-3, 1-5, and 5-1. respectively. ***B***, KINARM setup. ***C***, Time course of a baseline trial. The trial starts with an empty screen for 1000 ms. After this interval, the origin cue (1 cm blue circular cue) is shown on the bottom right part of the screen. When the end point (right-hand fingertip) enters the cue, the cue turns green, and the rectangular target and the distribution of reward are shown on the top left part of the screen. One second later, the origin cue turns white to indicate the GO signal. If the subject’s end point leaves before the GO is given, the screen turns blank and the trial is invalidated. When the end point enters the target, the rectangular cue turns green. The screen turns blank 500 ms after that. ***D***, Time course of a perturbed trial. The trial follows the same baseline trial pattern described in ***C***. However, the arm is perturbed perpendicularly to the straight line from the origin to the center of the target, 1–3 cm after the end point leaves the origin cue. Three separate factors are considered for perturbed trials: early/late, weak/strong, and right/left.

We introduce two hypotheses regarding changes of mind. First, we suggest that changes of mind are sensitive to the relative reward of the offers. To test this, we compared three bimodal distributions, one with identical rewards (3 vs 3) and two with different rewards (1 vs 5 and 5 vs 1) at the two extremes (Fig. 1*A*). We predicted changes of mind to occur more often in the former case, in which there is no advantage for reaching to either side. Second, we suggest that changes of mind are influenced by the momentary state of the motor system (i.e., velocity and distance from alternative targets when the perturbation occurs); to test this, we applied mechanical perturbations perpendicular to the direction of movement (left or right) in one-third of the trials. These were applied at two different levels of intensity (weak, 3 N; strong, 6 N), and at two different times during the movement. We implemented the early/late perturbation time by placing one of two distance thresholds at a distance from the origin (10% or 25% of the origin to target path length) to trigger the perturbation during probe trials.

The task consisted of 720 trials, performed in a single session. In each trial, the participant was asked to perform a reaching movement from a circular pale blue origin cue (diameter, 1 cm) to a wide rectangular blue target (width, 10 cm; depth, 1 cm), placed 15 cm away and rotated 135° counterclockwise (Fig. 1*A*). The location and rotation of the target were chosen to equalize the potential influence of motor costs for movements toward the right/left part of the rectangle, as this coincides with the direction of the arm maximal inertia (Hogan, 1985; Cos et al., 2011). In brief, movements toward either side of the target incurred approximately the same biomechanical cost. The goal for the participant was to maximize reward by aiming at specific positions along the long side of the rectangle. Since our goal was to assess the influence of reward expectations, at the beginning of each trial, the reward distribution was indicated to the subject using triangle displays for one of three bimodal distributions: 3-3, 1-5, and 5-1 (Fig. 1*A*). These distributions peaked at the right/left edges of the long edge of the rectangle and decreased toward zero when approaching its center. The distribution was zero off the right/left sides of the rectangle, implying that reaching movements missing the target would be awarded zero reward. The subject’s instruction was to freely select a reaching movement from the origin to any position along the long side of the rectangle, and the reward obtained on each trial was contingent on arrival position and the distribution of reward.

The session consisted of 720 trials of two types: two-thirds of baseline trials and one-third of probe trials, which were pseudorandomly interleaved. During baseline trials, subjects could perform their reaching movements without perturbation. During probe trials, the subject’s arm was mechanically perturbed in a direction perpendicular to the axis between the origin and the target sometime after the movement had been initiated and before the target had been reached. Each participant performed 10 blocks of 72 trials each in a single session (∼1 h and 15 min). Each block consisted of 48 baseline trials (16 baseline repetitions of each distribution of reward) and 24 probe trials, randomly interleaved. There were 24 types of probe trials, reflecting each combination of the distribution of reward (3-3, 5-1, or 1-5), the perturbation direction (PD; right or left), the intensity of the perturbation (weak/strong), and the time of perturbation (early/late). Each block contained one trial of each possible probe type (3 × 2 × 2 × 2 = 24). Trial order was randomized, and block order was counterbalanced across participants.

Real-time visual feedback of hand position was provided during the trial by a 1 cm white dot on the screen, synchronized with the tip of the participant’s right-hand position on the experimental table. The time course of each kind of trial is shown in Figure 1, *C* and *D*. A baseline trial began when the origin was shown on the screen and the subject entered the cue. Approximately 1 s later, the rectangular target and the distribution of reward were shown. After 500 ms, the distribution of reward was removed. After a 1 s interval, a GO signal was given when the origin cue vanished. The subject was instructed to perform a movement toward the position along the rectangle that they deemed most rewarding. If the subject left the origin before the GO signal was given, the experimental arrangement disappeared, and the subject had to wait until the regular trial duration of 7 s elapsed before resuming the next trial. Correct target entry resulted in the rectangle turning green. After 500 ms of holding position at the target, the target disappeared. This was followed by an intertrial interval of ∼1 s, the duration of which was dynamically calculated to obtain a fixed overall trial duration of 7 s, to prevent participants from performing faster movements just to increase reward rate. Note that although we did not provide an explicit measure of score or feedback after each trial, visual feedback of the end point was provided at all times. Subjects could therefore infer the reward attained at each trial just by checking their entry point along the rectangle side. The probe trials followed the same time course of the baseline trials, with the exception of the mechanical perturbation, which was applied when the end point had already moved over 10% or 25% of the total path length from the origin. At the beginning of each block, subjects were reminded that their goal was to maximize reward and that, during probe trials, they may have to change their mind to attain that goal.

### Statistical tests

#### Analysis of kinematics

**Figure 2.**
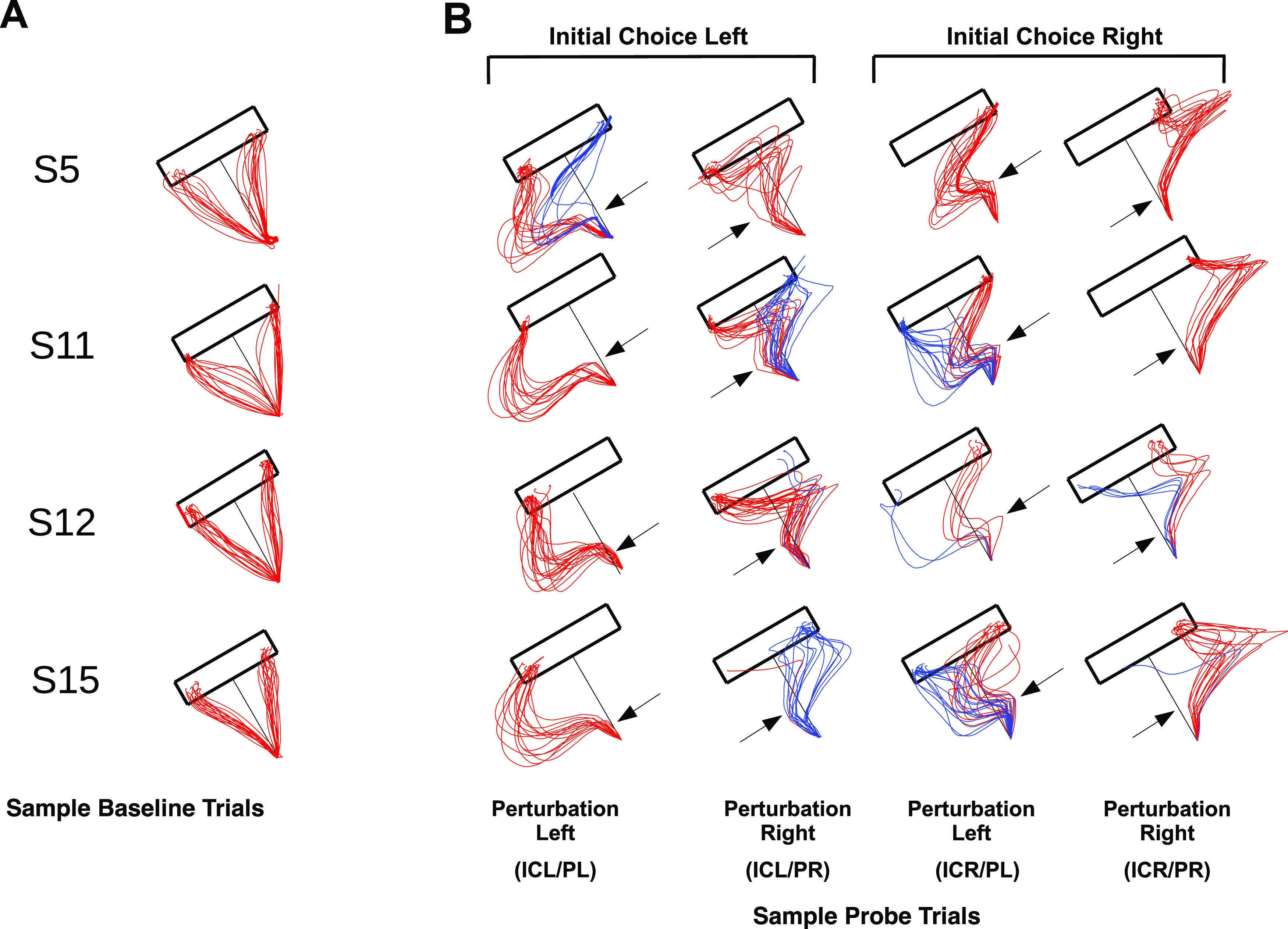
***A***, Typical baseline trajectories for subjects 5, 11, 12, and 15 during baseline trials. Please note that the figure shows trials from all three reward distributions: 3-3, 5-1, and 1-5. ***B***, Perturbed trajectories for subjects 5, 11, 30, and 33, classified as a function of (1) the initial choice direction (rightward/leftward) to the direction of motion and (2) the direction of the perturbation (rightward/leftward). Blue trajectories indicate CoM trials, while red trajectories indicate non-CoM trials (Extended Data Fig. 2-1).

Quantitative analyses of trajectories and velocities were performed with custom-written MATLAB scripts. First, we examined trajectories to determine the participant’s final choices, labeling them as right or left as a function of whether the movement end point lies on the right or left hemi-plane defined by the line between the origin and crossing the long rectangle side perpendicularly (Fig. 2*A*). We also labeled their initial choices as initial choice right (ICR) or initial choice left (ICL) as a function of whether the first 200 ms of the path trajectory lie mainly on the right or left side of that hemi-plane. We first characterized baseline trials during which subjects could freely choose their trajectories in the absence of perturbation, to gain an insight on the subjects’ kinematics in the absence of perturbation. Next, to study how subjects changed their mind, we performed a comparative analysis during probe trials, examining the initial movement direction as well as the final movement end point (Extended Data Fig. 2-1).

10.1523/ENEURO.0174-21.2021.f2-1 Figure 2-1 ***A***, Distribution of end point positions at arrival to the target as a function of reward distribution for each individual subject, during nonperturbed (baseline) trials: 3-3 (red), 5-1 (green), and 1-5 (blue). ***B***, Distribution of arrival positions as a function of reward distribution for each individual subject, during nonperturbed (baseline) trials: 3-3 (red), 5-1 (green), 1-5 (blue; Fig. 2). Download Figure 2-1, EPS file.

**Figure 3.**
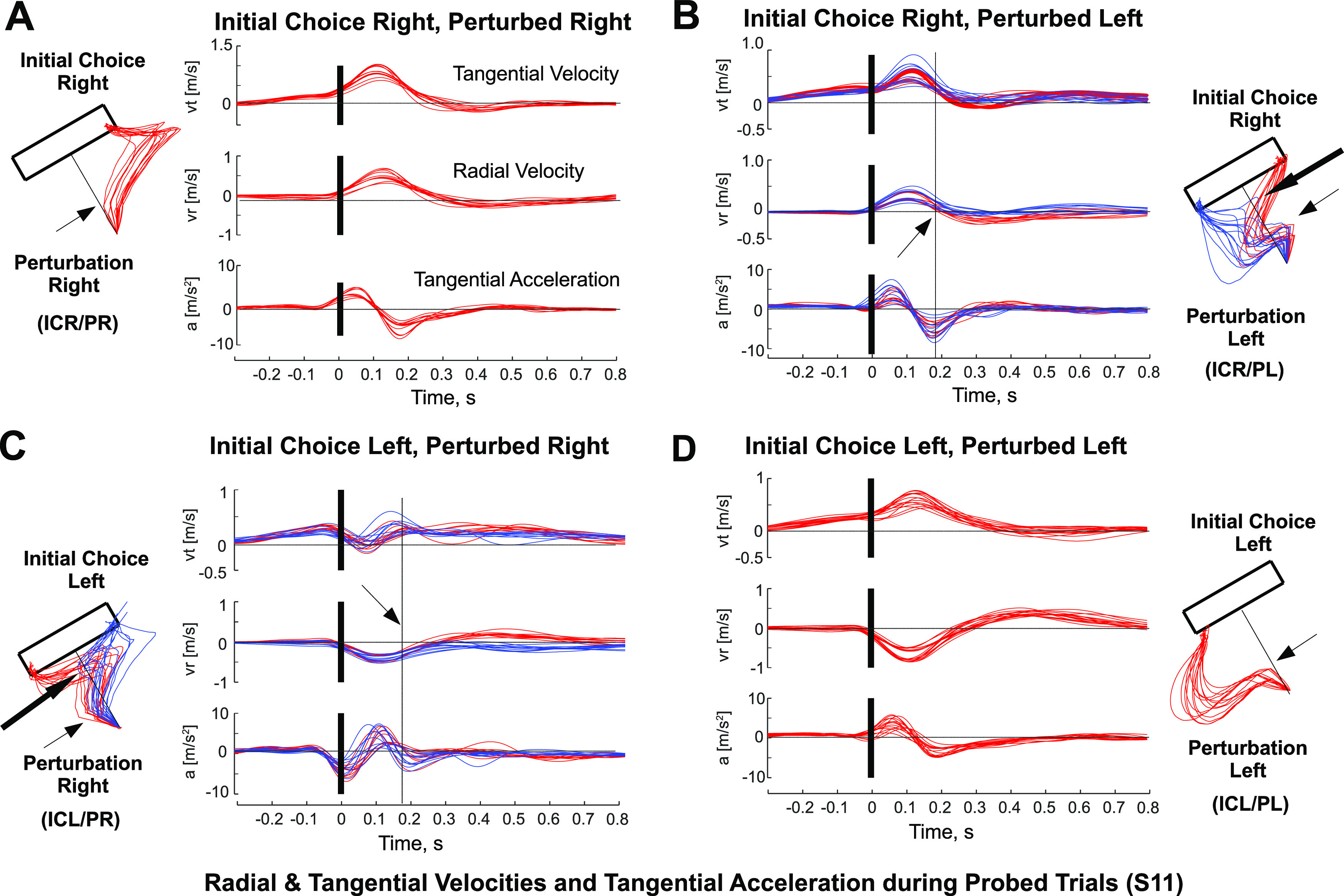
***A***, Case of ICR/PL. Perturbed radial velocities, tangential velocities, and tangential accelerations, aligned at the time of perturbation, indicated by a vertical black line, for four kinds of trials, using data from S11. The thin black arrow indicates the time at which the perturbation is applied. ***B***, Same as ***A***, but for initial choice right, perturbed left. The thick black arrow indicates the time at which the CoM comes into effect on the trajectory. ***C***, Same as ***A***, but for the case of initial choice left, perturbed right. ***D***, Same as ***A***, but for the case of initial choice left, perturbed left.

Four participants never changed their mind during probe trials and had to be discarded from further analysis about how changes of mind (CoMs) occurred. Hence, we used data from 12 participants for our analyses, and referenced their trajectories to the axis between the center of the origin cue and the middle of the long side of the target rectangle. The origin of the reference system is the center of the origin cue, with a positive *y*-axis in the direction toward the center of the long rectangle side, and a positive *x*-axis from the origin toward the right side of the rectangle and parallel to its long side (Fig. 1*A*). While the classification of baseline trials depended on their elected reaching target side alone (initial choice right/left), probe trials were classified, additionally, according to the time (early/late), intensity (strong/weak), and direction of the perturbation (perturbation left/right), and according to whether subjects changed their mind (Fig. 2*B*). To assess the effect of the perturbations on the subjects’ trajectories, we first grouped trajectories into those that shifted to the target side opposite to their initial choice (CoM) trajectories, and those that remained on the same side (non-CoM). Second, we characterized the state of the motor system by two complementary metrics: first the distances from each trajectory to the two lines defined between the origin and the bottom-right [distance right (DR)] and bottom-left [distance left (DL)] vertices of the rectangle long side (see Fig. 6*D*); and second, the radial and tangential velocities, as well as the tangential acceleration through differentiation for probe trials in each experimental case (Fig. 3). Again, we assessed the effect of the change of mind by subtracting the differences between the profiles during which there was a change of mind versus those in which there was no change of mind, for each type of probe trial.

#### Reward and probability of change of mind

We first characterized the statistics of the changes of mind by estimating the *p* parameter of a binomial distribution *B*(*n*,*p*) that would characterize the changes of mind, as a function of whether there was a CoM (potential CoM (PCoM) = 1) or not (PCoM = 0). To that end, we counted the number of times each subject changed target side over the total number of probe trials. The resulting binomial *p* parameter captures the probability of a CoM to occur.

We first estimated the binomial *p* parameter for each participant, and for each combination of the following four factors: ICR/ICL, perturbation direction [perturbation right (PR)/perturbation left (PL)], and the distribution of reward (3-3, 1-5, 5-1). Second, we performed a generalized linear model (GLM) to assess the influence of the R (−4, 0, 4) to be gained on PCoM, the PD (inward/outward), and their interaction. Group significance was calculated by running *F*/*t* tests across the β-regression coefficients obtained for each participant. For presentation purposes, we displayed those cases in which the *p* parameter between the even case (3-3) versus those cases in which opting for the target side opposite to their initial choice meant a large drop in predicted reward (5-1 or 1-5), as a function of whether the perturbation was PR or PL. Namely, we displayed the PCoM for the following four pairs of cases: ICR/PR, 3-3 versus 1-5; ICR/PL 3-3 versus 1-5; ICL/PR 3-3 versus 5-1; and ICL/PL 3-3 versus 5-1.

Furthermore, to analyze the dependence of the PCoM on reward to be gained from the PCoM itself and on the remaining experimental factors, we calculated the binomial *p* parameter for each participant and for each possible combination of R gain on CoM (−4, 0, 4), perturbation time (T; early/late), perturbation intensity (I; weak/strong), and velocity before the perturbation (V; slow/fast). T, I, and V were classified as early/late, weak/strong, and slow/fast using median splits within the distribution of T, I, and V of each participant. We then fitted a GLM to the resulting *p* binomial parameters against the four factors and their interactions for each individual participant. Group significance was established via Bonferroni-corrected *F*/*t* tests on each of the β-regression coefficients. Significance was established on whether the probability of the null hypothesis was <5% (*p* < 0.05). The effect of reward gain on the PCoM was fitted with a parametric sigmoidal function, as follows:

(1) $$\text{P(CoM})=\frac{e^{Q}}{1+e^{Q}};Q=a\times G+b,$$

where *G* is the reward to be gained from a CoM.

#### The temporal unfolding of the change of mind

To study the temporal unfolding of the changes of mind, we performed an analysis of CoM versus non-CoM trajectories during probe trials. Specifically, we focused our attention on differences of DR and DL trajectories (see subsection Analysis of kinematics; see Fig. 6*D*), between CoM and no-CoM trials, for each case of initial choice and perturbation direction: ICR/PR, CoM versus no-CoM; ICR/PL, CoM versus no-CoM; ICL/PR, CoM versus no-CoM; and ICL/PL, CoM versus no-CoM. For each participant, we aligned probe trial trajectories on the onset of movement and performed a sliding *t* test on the distance metrics DR and DL, between CoM and non-CoM trials, and calculated the two times along the path-trajectory at which CoM and non-CoM became significantly different with 95% and 99% probability.

We portrayed the temporal unfolding of the change of mind by means of two scatter plots of DR versus DL, sampled at two times of interest along the trajectory: at the point of peak deviation postperturbation, and at the time the difference between CoM and non-CoM trials in terms of DR (or DL) reached significance at *p* < 0.05. These scatter plots were also fitted with ellipses by means of principal component analysis, aligning their axes with the dimensions of maximum variability, with radii equal to the square root of the corresponding eigenvalues. These scatter plots also served the purpose of characterizing the state of the motor apparatus at any given time.

#### The state of the motor apparatus

In addition to assessing the influence of reward on the PCoM, we also performed an analysis of the influence of reward on movement by assessing the differences in DR and DL as a function of reward at the peak deviation. Namely, we predicted that reward would influence movements either by magnifying responses (increasing vigor; Guitart-Masip et al., 2011; Choi et al., 2014) or by increasing the subject’s concern for precision (diminishing arrival velocity; Trommershäuser et al., 2003). For this analysis, we selected only non-CoM probe trials, and classified them according to choice and perturbation direction: ICR/PR, ICR/PL, ICL/PR, and ICL/PL. Comparisons within each case were performed between trials in which the predicted reward was 3 and 5. The results are plotted separately for DR and DL as scatter plots and histograms as a function of reward case. Statistical significance between histograms was performed by means of a *t* test, and significance was established at *p* < 0.05. The analysis of the state is also complemented by quantifying the influence of movement velocity on the PCoM and time of Change of Mind (tCoM) as described in the previous subsections.

## Results

### Choice preferences

We first assessed the influence of reward on decision-making by calculating the following: (1) the target side preferences of each participant as a function of the reward distribution (3-3, 1-5, 5-1) during baseline trials; (2) the distributions of target arrival locations (Extended Data Fig. 2-1*A*); and (3) initial directions with respect to the midline defined by the coordinate system in Figure 1*A* for each reward distribution (Extended Data Fig. 2-1*B*). As expected, arrival distributions peaked around positions of the rectangle side that offered the largest amounts of reward for uneven distributions (1-5, 5-1), indicating that subjects’ choices were strongly biased by predicted reward. Furthermore, the initial directions showed that movements were most frequently directed toward those positions from the start.

### Analysis of kinematics

Figure 2*A* shows some typical baseline trial trajectories during 3-3 reward distributions for four subjects. Although the choice of target side is approximately as frequent toward the right or left side of the rectangle when the distribution is 3-3, some subjects exhibited mild lateral biases for one option over another (Extended Data Fig. 2-1*A*). Figure 2*B* shows typical trajectories for the same four subjects during probe trials. We classified probe trial trajectories as a function of their initial direction, perturbation direction (right/left), and CoM/non-CoM occurrence (see Materials and Methods). The task was designed to perturb with equal frequency toward the right and left side of the *y*-axis (Fig. 1*A*)—approximately perpendicular to the direction of movement so that subjects could not anticipate the upcoming perturbation or its direction. We predicted that perturbations toward the side of the target opposite to the subjects’ initial choice were likely to yield a change of mind after the onset of movement. However, in addition to confirming this prediction (Fig. 2*B*, S11, S16), our results also show that a few changes of target side occurred when both the initial choice and the perturbation directions matched (Fig. 2*B*; S5, initial choice left/perturbation left; S13 and S16, initial choice right/perturbation right), possibly as a result of the reflex that results from the perturbation.

Figure 3 shows the tangential velocity and acceleration, and radial velocity traces for four types of probe trials, for a typical subject (S11). We aligned the traces at the time of perturbation. The four types of trials are as follows: ICR/PR (Fig. 3*A*), ICR/PL (Fig. 3*B*), ICL/PR (Fig. 3*C*), and ICL/PL (Fig. 3D). A first visual analysis suggests that the first moment of divergence between non-CoM and CoM trajectories occurs ∼170–80 ms postperturbation (Fig. 3*B*,*C*). Incidentally, this is approximately aligned with the time of peak deceleration.

### Reward and changes of mind

**Figure 4.**
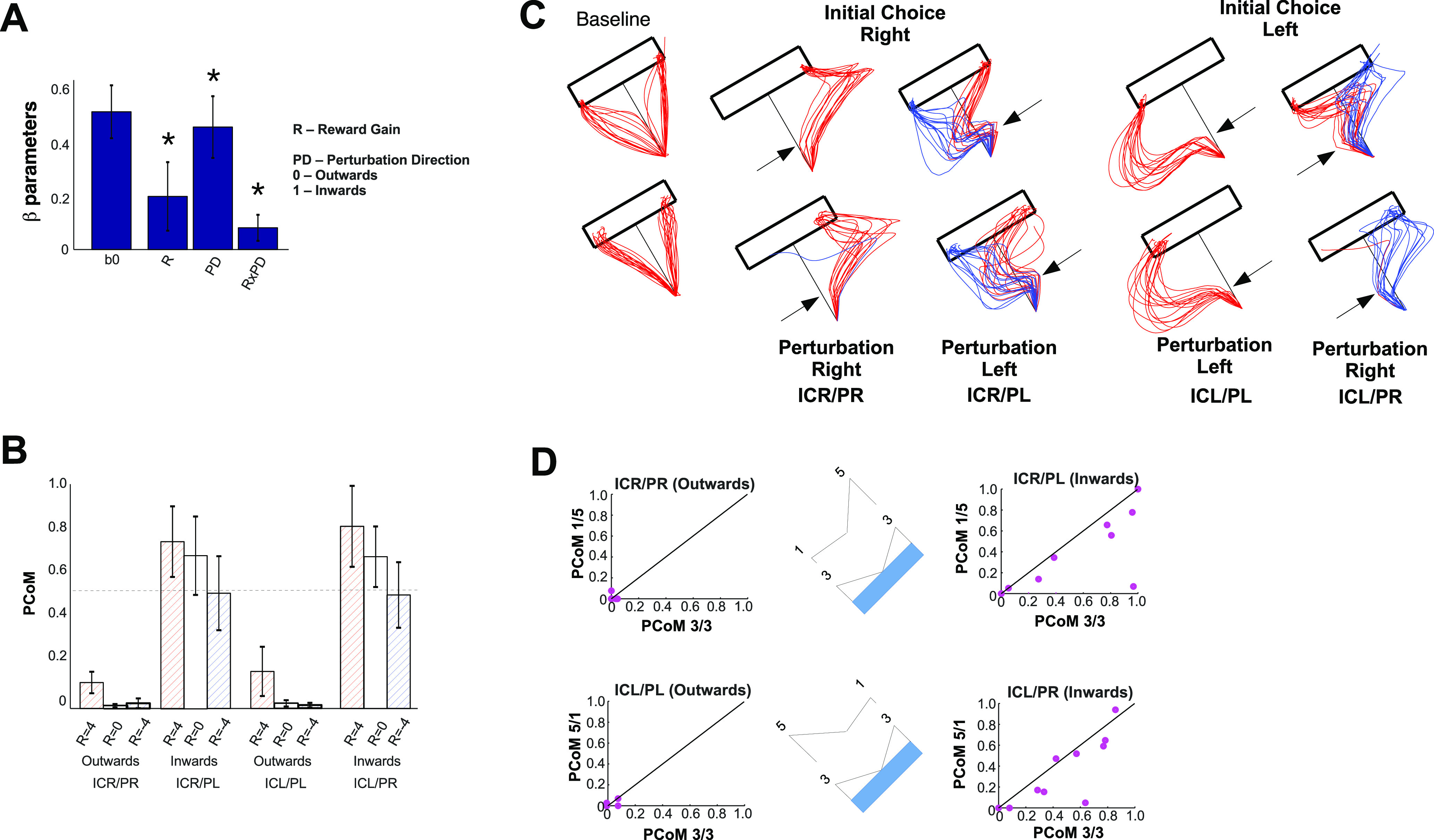
***A***, Group average and SEM regression coefficients on the PCoM as a function of reward gain (−4, 0, 4), perturbation direction (inward/outward), and their interaction. ***B***, Group average and SEM PCoM as a function of reward, perturbation direction, and initial choice. ***C***, Baseline and probe trial trajectories (Fig 3) for two subjects. ***D***, Scatter plots of the average PCoM per subject when aiming at R = 5 versus R = 3 in four cases: ICR/PR 3-3 versus 1-5; ICR/PL 3-3 versus 1-5; ICL/PR 3-3 versus 5-1; ICL/PL 3-3 versus 5-1.

CoMs may occur when the perturbation assists the movement toward the target side opposite to their initial choice and may be biased by the distribution of reward at that trial. To analyze the dependence of CoMs on reward and perturbation direction, we estimated the PCoM as a binomial random variable, by first fitting the *p* parameter for each participant and experimental case, as defined by the following two factors: the R to be gained from PCoM and the PD with respect to the initially selected one (inward/outward). We then fitted a GLM on those binomial *p* parameter values for each participant with factors R, PD, and R × PD (see Materials and Methods). The group average and SD β-regression coefficients are shown in Figure 4*A*, reporting a strong influence of the perturbation direction (*F*_(1,12)_ = 48.32, η^2^ = 0.80, *p* = 1.5E-5, Cohen’s *d* = 4.01), strongly favoring the inward perturbation direction; a positive effect of reward (*F*_(1,12)_ = 12.3; η^2^ = 0.51, *p* = 0.0045, Cohen’s *d* = 2.04), and a marginal interaction between both (*F*_(1,12)_ = 6.8; η^2^ = 0.36, *p* = 0.023, Cohen’s *d* = 1.5). Figure 4*B* shows the group average PCoM, including the potential reward gain, the initial direction of movement, and the direction of perturbation, showing that both the direction of perturbation and reward exert a significant influence on the PCoM. To specifically display the influence of the direction of perturbation, Figure 4*C* shows some typical trajectories, displayed as a function of each initial choice and PD (ICR/PR, ICR/PL, ICL/PL, ICL/PR). Again, inward PDs (ICR/PL and ICL/PR) were the most likely to yield a CoM. For presentation purposes, Figure 4*D* shows the PCoM per participant, as a function of reward distribution (1-5 vs 3-3 or 5-1 vs 3-3). In brief, it shows that because the 3-3 condition implied no potential loss of reward, CoMs were more likely to occur in that distribution. In other words, the effect of reward on the PCoM becomes evident from the fact that subjects change their minds more frequently in the 3-3 condition, when a CoM implies no loss, and less frequently in the 1-5 and 5-1 conditions, where a CoM implies a loss (by assuming that subjects initially selected the side with the higher reward, which was almost always the case in our data). In summary, CoMs are most likely to occur during 3-3 trials and when perturbed inward.

**Figure 5.**
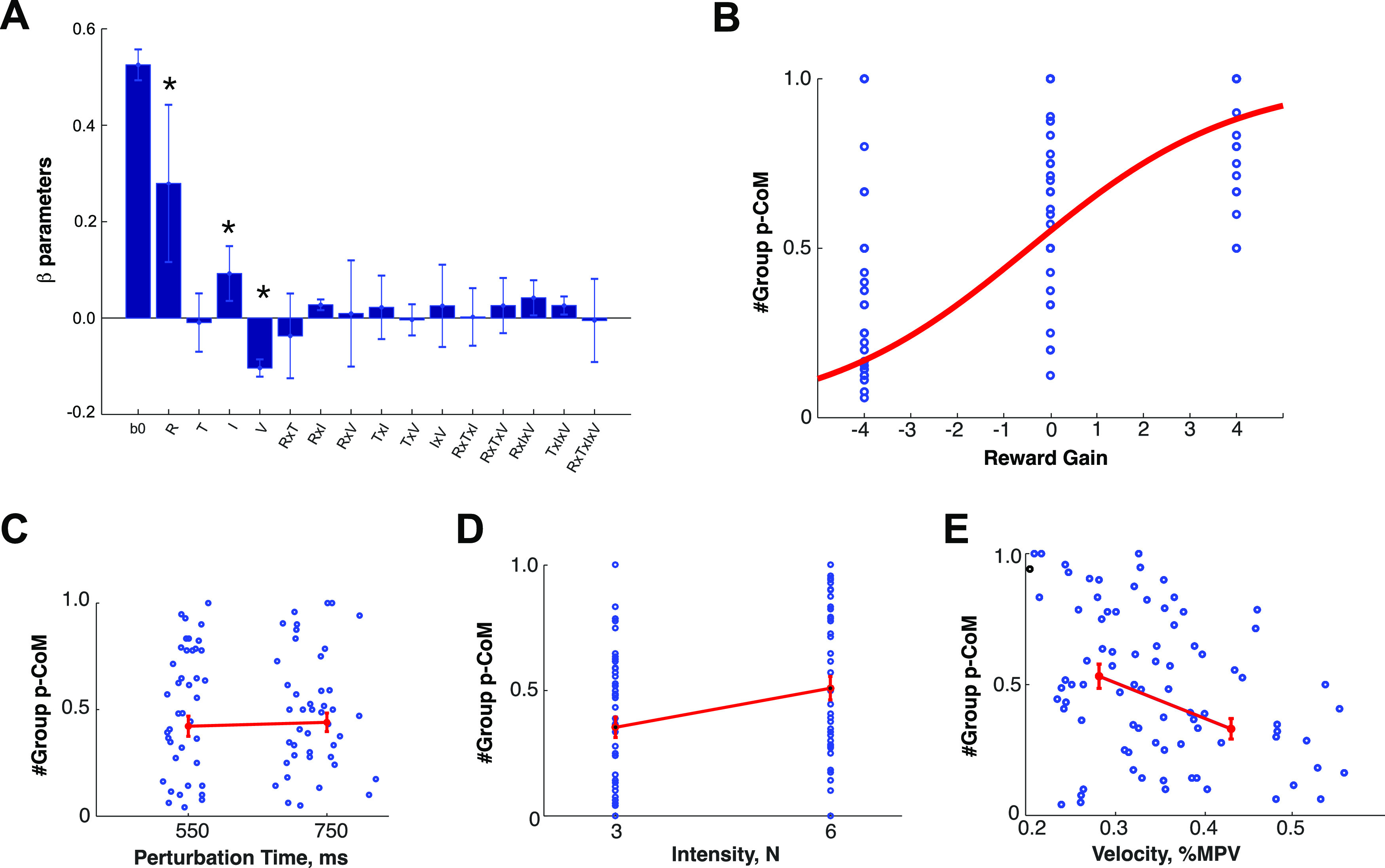
***A***, Group average regression coefficients a GLM on the *p* binomial distribution parameter, calculated on an individual subject basis, as a function of the following four factors: reward to be gained from PCoM, time of perturbation, intensity of perturbation, tangential velocity, and their interactions.Group significance is reported by a * symbol (*p*<0.05). ***B***, Group average effect of reward gain on PCoM. ***C–E***, Group average effects for the perturbation time, perturbation intensity and velocity at the time of perturbation. Error bars indicate the SEM.

In addition to this, we also assessed the dependence of PCoM on the reward to be gained by a CoM, and on non-explicit factors—unknown to the subject at the time of the trial, such as the direction of the perturbation, its intensity, and the hand velocity before the perturbation. To this end, we fitted a second GLM to the binomial *p* parameter values obtained per participant, and calculated for each combination of the aforementioned factors (Fig. 5*A–E*). Again, group significance was obtained for reward gain (*F*_(1,12)_ = 14.44; η^2^ = 0.55, *p* = 0.0025, Cohen’s *d* = 2.21), perturbation intensity (*F*_(1,12)_ = 31.81; η^2^ = 0.73, *p* = 0.00012, Cohen’s *d* = 3.29), and velocity (*F*_(1,12)_ = 151.23; η^2^ = 0.93, *p* = 9.80E-6, Cohen’s *d* = 7.29), but not for the time of perturbation (*F*_(1,12)_ = 0.20; η^2^ = 0.02, *p* = 0.70, Cohen’s *d* = 0.28; Fig. 5*C*). There were no significant interactions (*p* > 0.05). In summary, changes of mind were more likely to occur when there was reward to gain (sigmoid coefficients a = 2.24, b = 0.20; mean square error = 0.12; Fig. 5*B*) after strong perturbations (Fig. 5*D*) and during slow movements (Fig. 5*E*), but not for early versus late perturbations (Fig. 5*C*).

### State of the motor system

**Figure 6.**
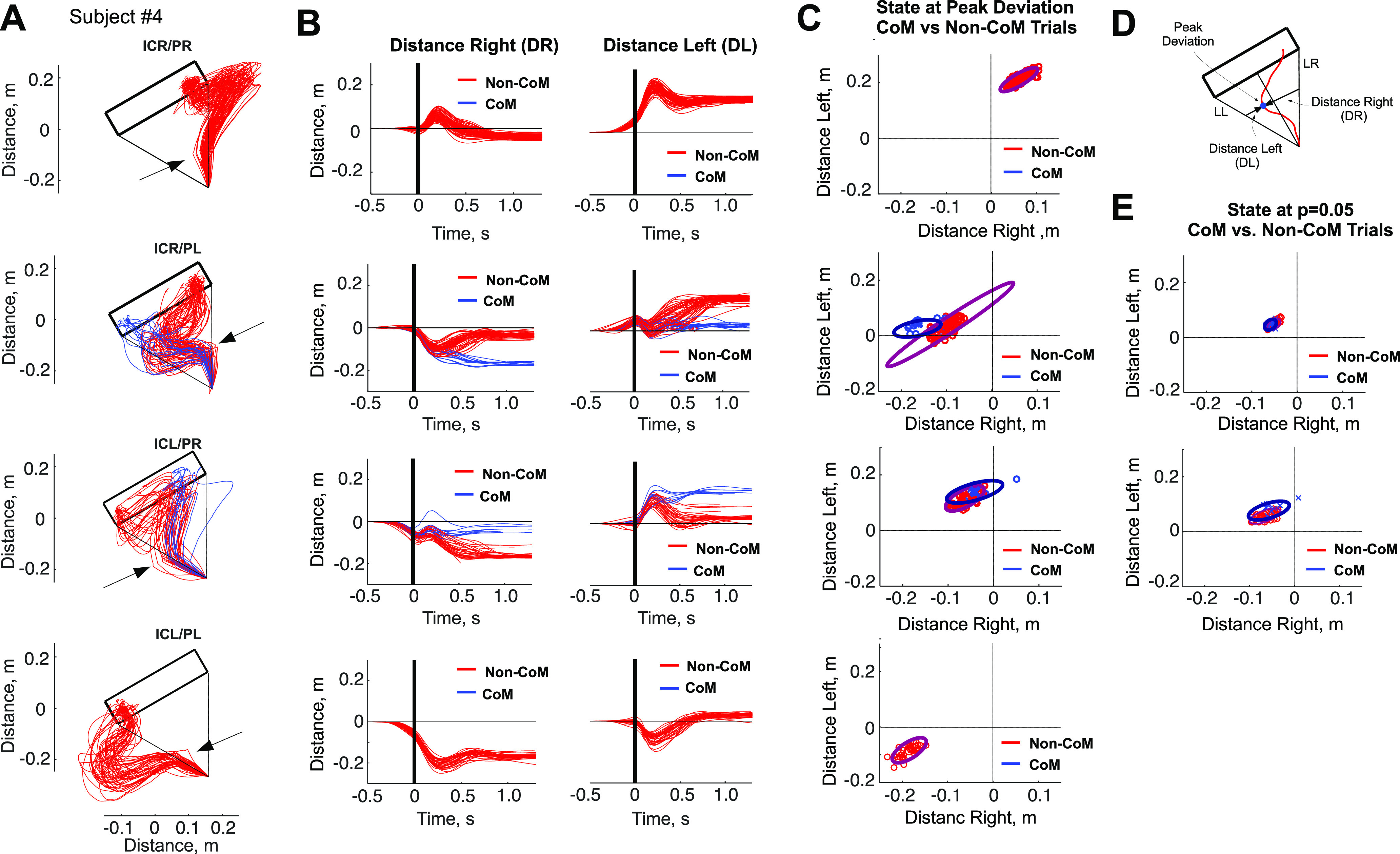
***A***, Probe trajectories for the cases of study: ICR/PR, ICR/PL, ICL/PR, and ICL/PL for subject 4. ***B***, Distance right and left in each of the four cases shown in ***A***. Blue, CoM trajectories; red, non-CoM trajectories. ***C***, State of the motor apparatus, characterized by DR and DL at the time of peak deviation, and at the time the difference between CoM and non-CoM trajectories became significant. ***D***, Geometrical definitions for the distance-based analysis of the state of the motor apparatus: DL/DR for a sample perturbed trajectory. Illustration of the trajectory peak deviation. ***E***, State of the motor apparatus, characterized by DR and DL at the time they became statistically significant with *p* = 0.05 (Extended Data Figs. 6-1, 6-2, 6-3, 6-4).

Our characterization of the state of the motor apparatus and its influence on the changes of mind encompasses two complementary metrics. The first is the end point velocity, which captures the dynamics of the motor apparatus at the time of perturbation, part of the PCoM analysis of the previous section. The second is the distance between each probe trial trajectory to the two lines used as reference axes for movements toward either rectangle side (from the origin to the right/left bottom vertices of the rectangle target, DR and DL, respectively; see Materials and Methods). Red and blue traces in Figure 6*A* and Extended Data Figures 6-1, 6-2, 6-3, and 6-4 show the resulting path distances for four typical subjects (S4, S5, S11, and S32), alongside with the subject end point trajectories, classified as a function of the initially aimed target side (right/left), the direction of perturbation (right/left), and CoM/non-CoM.

10.1523/ENEURO.0174-21.2021.f6-1 Figure 6-1 ***A***, Probe trajectories for the cases of study: ICR/PR, ICR/PL, ICL/PR, ICL/PL for Subject 5. ***B***, DR and DL in each of the four cases shown in ***A***. Blue, CoM trajectories; red, non-CoM trajectories. ***C***, State of the motor apparatus, characterized by DR and DL at the time of peak deviation, and at the time the difference between CoM and non-CoM trajectories became significant. ***D***, State of the motor apparatus, characterized by DR and DL at the time they became statistically significant with *p* = 0.05 (Fig. 6). Download Figure 6-1, EPS file.

10.1523/ENEURO.0174-21.2021.f6-2 Figure 6-2 Same as Figure 6-1, for S11 (Fig. 6). Download Figure 6-2, EPS file.

10.1523/ENEURO.0174-21.2021.f6-3 Figure 6-3 Same as Figure 6-1, for S16 (Fig. 6). Download Figure 6-3, EPS file.

10.1523/ENEURO.0174-21.2021.f6-4 Figure 6-4 The *p* value of the *t* test calculated between trajectories in which each subject changed his/her mind and the trajectories in which the subject stuck to his/her original choice after a perturbation. The comparison was performed by first calculating the distance between each trajectory and the straight path was defined between origin and the right or left bottom vertex of the target. The *t* test is calculated at each time step between both distributions of distances and is calculated for each subject individually. ***A***, *p* Values as a function of time for each individual subject. ***B***, Time in milliseconds at which the *p* value for each subject became significant at 0.05 (Fig. 6). Download Figure 6-4, EPS file.

Although the context in which the CoM may occur is generated by a mechanical perturbation that elicits a motor reflex in the opposite direction, the subsequent trajectory toward the opposite side of the target must be voluntarily enacted. Our hypothesis is that the gating of the change of mind depends on the state of the motor system at the time the perturbation. If this were the case, we should observe significantly different states of the motor system in correspondence to perturbations that elicited CoMs versus those that did not. To test this, we measured the joint (bidimensional) path distances from each trajectory to the two lines defined between the origin and the bottom right (DR) and bottom left (DL) vertices of the long side of the rectangle (Fig. 6*D*), at the time of peak deviation. Figure 6*C* and Extended Data Figures 6-1*C*, 6-2*C*, 6-3*C*, and 6-4*C* show this bidimensional proxy of the state, plotted as *x*–*y* planar coordinates, for CoM (blue) and non-CoM (red) trials. We used an enveloping ellipse to capture data covariance. Since changes of mind were primarily elicited in trials where the direction of the perturbation and the target side initially aimed for were opposite, we restricted the CoM versus non-CoM comparison with those cases. We established the critical time at which both sets of trajectories diverged from each other by means of a sliding *t* test at each 10 ms between CoM versus non-CoM DR/DL trajectories, from the onset of movement until the peak deviation (Fig. 6*D*, yellow and red traces; see Materials and Methods). Remarkably, all subjects exhibited significant differences before peak deviation (*p* < 0.05), varying from 30 to 450 ms (average, 153 ms; Fig. 6*D*, Extended Data Fig. 6-4).

### The effect of reward on the state of the motor system

**Figure 7.**
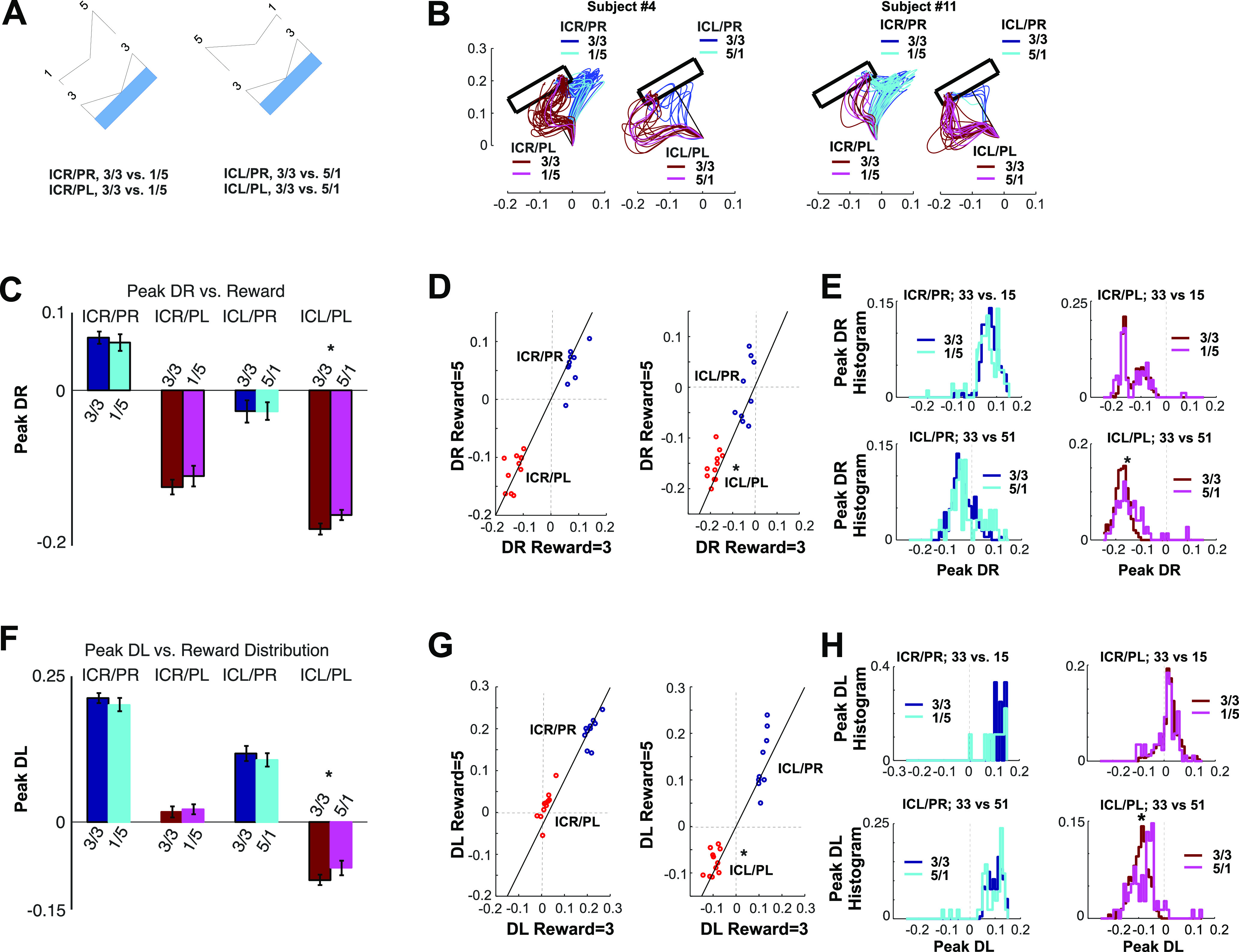
Effects of reward on DR and DR at peak deviation on non-CoM probe trials in four cases listed next. ***A***, ICR/PR, 3-3 versus 5-1; ICR/PL, 3-3 versus 5-1; ICL/PR, 3-3 versus 1-5; ICL/PL, 3-3 versus 1-5. ***B***, Sample trajectories for the cases described in ***A*** for subjects 4 and 11. ***C***, Group average and SE of the peak DR for the cases described in ***A***. ***D***, Scatter plot of each subject’s average DR, evaluated at R = 5 versus R = 3 trials. ***E***, Group distributions of peak DR, evaluated in the cases described in ***A***. ***F–H***, Same as ***C–E***, but for DL.

We hypothesized that state of the motor apparatus, characterized by the relative distances DR and DL, should vary as a function of predicted reward, making CoMs more likely to occur during 3-3 trials. To test this, we quantified the influence of reward on the state of the motor system and assessed whether larger rewards yield faster movement initiations and longer deceleration phases toward the target. To this end, we compared our bidimensional path distance metric (DR and DL) between non-CoM-probe trajectories only and restricted our analysis to the cases in which target positions offering a reward of 3 (3-3) versus those aimed at a reward of 5 (5-1 or 1-5). Specifically, we performed the following four comparisons: ICR/PR, 3-3 versus 1-5; ICR/PL, 3-3 versus 1-5; ICL/PR, 3-3 versus 5-1; and ICL/PL 3-3 versus 5-1 (Fig. 7). Their related trajectories are shown in Figure 7*B* for subjects 4 and 11.

Figure 7*C* shows the group average and SE of DR at the time of peak deviation, across probe trajectories for the four aforementioned comparisons. Figure 7*D* shows two scatter plots of the mean DR per participant as a function of predicted reward (5 vs 3). If reward were not to exert any effect on the state, we should expect DR to assume equal values regardless of reward prospect, to align with the dashed diagonal in Figure 7*D*, and to obtain equal distributions of peak DR values for the four cases (Fig. 7*E*). Figure 7*F–H* shows the equivalent comparisons for DL.

To test for significance, we performed a set of GLMs on the peak DR and DL, with respect to the reward to be gained from a CoM for each of the four cases of interest (Fig. 7). At each case, we fitted a GLM to the data from each subject and performed a *t* test across the coefficients obtained across groups (Fig. 7). Although Figure 7, *C–E* and *F–H*, shows trends consistent with that hypothesis, statistical tests reached statistical significance only in the case of initially aiming toward the left and being perturbed toward the left (ICL/PL; Fig. 7*D*,*G*, bottom right scatter plot; *F*_(1,12)_ = 9.15, η^2^ = 0.45, *p* = 0.010, Cohen’s *d* = 1.81). In brief, for the case ICL/PL, the DL was significantly smaller when aiming at 5 than when aiming at 3. The shift exerted by the perturbation is slightly smaller when the prospect of reward is 5 versus 3, strongly suggesting that the commitment is stronger whenever the participant is certain that there is no other more desirable option. While a similar trend is also visible in the ICR/PL and ICL/PR cases, the effect remained marginally nonsignificant (*F*_(1,12)_ = 3.60, η^2^ = 0.23, *p* = 0.082, Cohen’s *d* = 1.09; *F*_(1,12)_ = 2.4, η^2^ = 0.17, *p* = 0.16, Cohen’s *d* = 0.91, respectively).

## Discussion

Traditional models describe decision-making as a sequential process of deliberation followed by commitment, all of which precede the planning and execution of the chosen action. However, in ecologically valid conditions, it is often useful to initiate action before the deliberation phase is complete, and to revise the initial plan along the way, as a consequence of, for example, changing prospects or motor costs (Cisek and Pastor-Bernier, 2014; Lepora and Pezzulo, 2015; Domínguez-Zamora and Marigold, 2019; Grießbach et al., 2021; Pierrieau et al., 2021). This embodied choice perspective suggests that the deliberation process has to remain flexible even after action initiation, to permit re-evaluating the initial choices—and “changing mind” when necessary. While changes of mind during action execution have been observed empirically (Resulaj et al., 2009; Song and Nakayama, 2009; Barca et al., 2012), it is not clear whether they reflect a truly deliberative process, and whether they are sensitive to economic rewards (i.e., the reward of the selected and unselected offers) and situated aspects of the choice (e.g., the state of the motor system during the choice and the costs of revising the initial plan).

Here we studied these economic and motor-related determinants of changes of mind during decision-making, by exploiting the fact that changes of mind can be triggered externally by perturbing the motor apparatus during the choice (Nashed et al., 2014). We designed a task in which participants had to select a reaching path trajectory from an origin to a wide rectangular target, where the reward was distributed nonuniformly as a function of the arrival end point. Rewards could be even (3-3) or uneven (1-5/5-1) at the two target sides. Critically, we applied mechanical perturbations after the choice was made, at different phases during the movement and in different directions, sometimes toward the lower-reward side of the target.

Our results show that, as expected, participants facing a choice between two target sides offering different rewards initially selected the direction of movement offering the highest prospect, whereas participants facing a choice between two regions offering the same prospect made their selection with approximately equal frequency. After a perturbation was applied, participants altered their initially selected target side most frequently when the change did not result in a reduction of reward, the perturbation was more intense, the movement was slower, and the distance from the initially selected target was greater. These results indicate that changes of mind were influenced both by the predicted reward associated with the action and the state of the motor system at the time of the perturbation.

These findings have two main implications. First, they provide supporting evidence for the notion that deliberation continues and remains flexible after movement onset, possibly because unselected potential actions are not completely suppressed or discarded (Lepora and Pezzulo, 2015). From a neurobiological perspective, the affordance competition hypothesis suggests that situated choices are resolved through a biased competition between neuronal populations corresponding to potential actions that implement the competing choices, as observed in the monkey premotor cortex (Cisek, 2007; Cisek and Kalaska, 2010; Pezzulo and Cisek, 2016). Our findings suggest that when there is a chance to revise the initial decision, the competition between potential actions is not completely settled before action initiation, possibly because the initially unselected neuronal population retains some subthreshold activation, and can take control afterward.

Second, our findings suggest that decision-makers never completely commit to their initial choice during situated decisions. Rather, they have a variable degree of commitment to their initial choice, which depends on the relative reward of the offers and on the state of the motor system. The pattern of results we observed suggests that the commitment to the initial choice is weaker when the decision is between even-rewarded choices, which makes changes of mind more likely. Furthermore, the commitment is stronger if the initially selected action leads to higher rewards, which makes the initial choice more resistant to external perturbations. Furthermore, our results also suggest that for a change of mind to occur, the state of the motor system has to be within a specific range, and that the external perturbation can act as a trigger for a change of mind only when departing from those states. Note that one can interpret the state of the motor system both as a reflection of a centrally computed degree of commitment (e.g., because one is committed, one decides to move faster) or as a cause of commitment (e.g., the action can be fast purely because of variability, but if it is fast, it is less prone to changes of mind). These two hypotheses remain to be disentangled in future studies.

More generally, our findings fit within a growing body of work suggesting that during natural behavior, decision-making and movement planning unfold together, in an integrated fashion, within highly distributed circuits spanning what have traditionally been considered purely cognitive versus sensorimotor regions of the brain (Shadlen et al., 2008; Cisek and Kalaska, 2010). Of course, in some conditions, such as abstract decisions between stable options without the pressure to act (e.g., choosing a chess move, deciding about a house to buy), the processes of outcome valuation and action control will occur at very different moments in time, each engaging only a restricted subset of the relevant neural mechanisms. Nevertheless, the organization of these mechanisms evolved for a different type of situation, regularly encountered during natural behavior (Cisek, 2019), in which animals must make decisions even during ongoing sensorimotor activity.

## Synthesis

Reviewing Editor: Alexander Soutschek, Ludwig-Maximilians-Universität München Decisions are customarily a result of the Reviewing Editor and the peer reviewers coming together and discussing their recommendations until a consensus is reached. When revisions are invited, a fact-based synthesis statement explaining their decision and outlining what is needed to prepare a revision will be listed below. The following reviewer(s) agreed to reveal their identity: Giuseppe Pellizzer. Note: If this manuscript was transferred from JNeurosci and a decision was made to accept the manuscript without peer review, a brief statement to this effect will instead be what is listed below.

Both reviewers agree that the quality of the manuscript has improved compared with the previous version they had reviewed, but several issues still remain open. One key issue is the t-test comparing the inward and outward perturbation conditions (p.8 - see comment 3 of reviewer 1), but also the nature of the rewards on the behavioral task remains unclear - were the accumulated rewards paid out as bonus to the participants? If the rewards were only hypothetical in nature, it seems not appropriate to state that the task measures “reward-driven decision making” (p.5). It is also important that the authors report more details for their statistical tests (t/F values, degrees of freedom, and effect sizes measures in addition to p-values; see comment 4 of reviewer 1).

Reviewer 1:

This is a manuscript that I had reviewed for JNsc and that was transferred to eNeuro. The authors revised the manuscript and responded to all the comments. I thank the authors for the clarifications, and/or revisions to the points raised in the comments. However, I think that there are several points that remained insufficiently clarified or revised. Here I just mention those points.

1. I had raised a question regarding the nature of the reward. It seems to me that for a reward-based decision-task it is a rather basic requirement to know what the reward was. The authors replied that it was ‘was determined as a combination of the arrival position and the distribution shown in the screen’, and that it was an ‘abstract notion’, not linked to a monetary compensation. I understand how it was determined (that was not the question), and I am fine with an abstract reward, but this still does not tell us what information the subjects received regarding that ‘abstract’ reward? What feedback about the level of reward did they get? Were they given a score? Did they get any feedback? Was it just their observation of where the cursor landed on the target that was considered to be the reward? However, it seems that there must have been some form of feedback other than the cursor since it is written that the trials had a ‘fixed overall trial duration of 7s, to prevent participants from performing faster movements just to increase reward rate”. If no feedback was given, why would the reward rate matter? A reader should not have to guess all of these things. More explicit clarity about this simple point is needed.

2. I had made a comment about what looks like an inconsistency in the examples provided for the distribution of choice side (Fig. 2A and Suppl. Fig. 1A), and the response of the authors did not really address it. Specifically, I was referring to the example of S13 in these two figures (although the same issue may be valid for other subjects). The text on p. 8 states that Fig. 2A shows typical baseline trajectories in the 3-3 condition. The legend does not specify that it is just the 3-3 condition. In Fig. 2A, we see that the trajectories of S13 are pretty much equally distributed left and right in the baseline 3-3 condition. However, when one looks at Suppl. Fig. 2A, the histogram of the target selected by S13 in the baseline 3-3 condition (red histograms) show that most trajectories were to the left and very rarely to the right. So, my question was how is it that one figure shows a fairly balanced number of choices toward left and right (Fig. 2A), and the other shows a clear imbalance in those choices (Suppl. Fig. 1A)? My understanding is that these two figures represent essentially the same data, and that the distribution for S13 of left versus right choices should be consistent in these two figures. If they are not representing the same data then something needs to be corrected or clarified in the text and/or legend.

3. I had asked to clarify the test used for the distribution of CoM in Fig. 4C. In my view from classic statistics, the analysis of a hypothesis by doing separate analyses on subsets of data of the same experiment is not okay since that does not take into account the overall variance within and across conditions. I am not a statistician, but that is my understanding on the matter. Regardless of that issue, in addition it remains that it is not clear how the analysis (t-test) was done, since the data for four comparisons (see p. 8 Reward and changes of mind) were somehow combined in only two t-tests. For example, the comparison of the “Inwards” conditions includes the data of ICL/PR 3-3 against 5-1 and those of ICR/PL 3-3 against 1-5. How can this become a single t-test? My guess is that the data of the 12 subjects for those two comparisons were put together for a single t-test which means that it would be like having done the analysis using data of 24 independent subjects, which would be not be acceptable. It could also be that the authors computed an average PCoM per subject across the two Inwards comparisons to keep a N=12, but since how the data were combined was not indicated, and neither the t-test result and its degrees of freedom, one cannot be not sure of what was done exactly.

4. For the results of the GLM analysis on p. 8, the specifics of the F test are missing for the effect of reward gain. For the test reported on p. 9, and may be others that I have missed, it is generally no longer considered acceptable to just indicate p=0.012 without indicating the test used, the result of the test, and other information that may be pertinent, like degrees of freedom, etc. The Information for Authors on the JNsc website provides links detailing the information that most Journals nowadays require for presenting statistical results.

5. I had indicated that there was a potential graphical issue in Fig. 2 with arrows representing the direction of perturbation that are plotted in different sizes. Although the authors indicated that they corrected it, that issue is still present.

6. I had indicated that the units of velocity in Fig. 3 needed to be corrected to [m/s]. The authors answered that they made the correction, but they didn’t. The error is still there.

7. I had signaled that some trajectories were drawn “fatter” than others; those are still there. There are several cases in Fig. 2B and one in Perturbation right/ICL/PR of Fig. 4A.

8. I had mentioned to indicate what are the error bars in Fig. 5 (SEM, CI, etc.). The authors responded that they included that information in the legend. I cannot find it.

Reviewer 2:

I was a reviewer (Reviewer #2) on this manuscript for a previous journal. The authors have done an reasonable job of responding to my previous comments and concerns with their revision. I still have a few more comments though.

I noticed that in their new Fig. 3, the authors did not make the correction they said they made -- the authors need to swap vr and vt y-axes to correspond to the bolded text in the figure.

Also, I am still confused on the color-coding on the single-subject data points in Fig. 4B (blue and red) and how these correspond to the blue and red traces in Fig. 4A. Please clarify this more. Throughout the paper you have blue trajectories indicate COM trials, and it isn’t obvious how this color-mapping applies to the single subject data points.
